# Supplementary material for: Mutations in PCYT2 disrupt etherlipid biosynthesis and cause a complex hereditary spastic paraplegia
Source: Brain. 2019 Oct 22;142(11):3382–97. doi: 10.1093/brain/awz291 (PMC6821184; doi:10.1093/brain/awz291)
Supplement: awz291_Supplementary_Data [file awz291_supplementary_data.zip › awz291-Suppl_data/Supplementary_Data4.zip!/Supplementary information 4 - plasma lipidomics.zip!/Plasma lipidomics/Control vs Patient2/Statistical plots and data/SM[d] species/Heatmap.pdf]

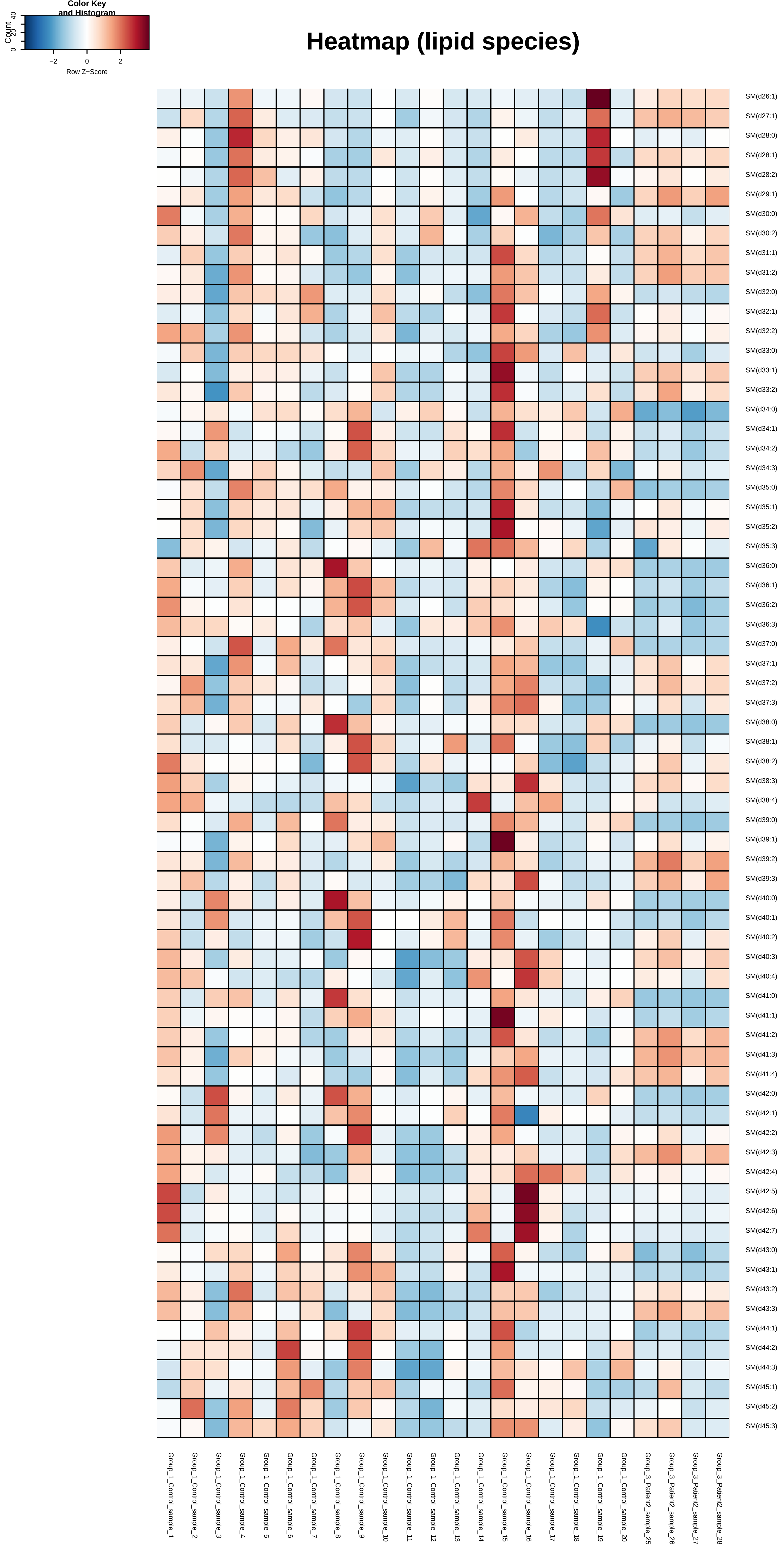
Color Key
and Histogram
Count
0
20
40
−2
0
2
Row Z−Score
Heatmap (lipid species)
SM(d26:1)
SM(d27:1)
SM(d28:0)
SM(d28:1)
SM(d28:2)
SM(d29:1)
SM(d30:0)
SM(d30:2)
SM(d31:1)
SM(d31:2)
SM(d32:0)
SM(d32:1)
SM(d32:2)
SM(d33:0)
SM(d33:1)
SM(d33:2)
SM(d34:0)
SM(d34:1)
SM(d34:2)
SM(d34:3)
SM(d35:0)
SM(d35:1)
SM(d35:2)
SM(d35:3)
SM(d36:0)
SM(d36:1)
SM(d36:2)
SM(d36:3)
SM(d37:0)
SM(d37:1)
SM(d37:2)
SM(d37:3)
SM(d38:0)
SM(d38:1)
SM(d38:2)
SM(d38:3)
SM(d38:4)
SM(d39:0)
SM(d39:1)
SM(d39:2)
SM(d39:3)
SM(d40:0)
SM(d40:1)
SM(d40:2)
SM(d40:3)
SM(d40:4)
SM(d41:0)
SM(d41:1)
SM(d41:2)
SM(d41:3)
SM(d41:4)
SM(d42:0)
SM(d42:1)
SM(d42:2)
SM(d42:3)
SM(d42:4)
SM(d42:5)
SM(d42:6)
SM(d42:7)
SM(d43:0)
SM(d43:1)
SM(d43:2)
SM(d43:3)
SM(d44:1)
SM(d44:2)
SM(d44:3)
SM(d45:1)
SM(d45:2)
SM(d45:3)
Group_1_Control_sample_1
Group_1_Control_sample_2
Group_1_Control_sample_3
Group_1_Control_sample_4
Group_1_Control_sample_5
Group_1_Control_sample_6
Group_1_Control_sample_7
Group_1_Control_sample_8
Group_1_Control_sample_9
Group_1_Control_sample_10
Group_1_Control_sample_11
Group_1_Control_sample_12
Group_1_Control_sample_13
Group_1_Control_sample_14
Group_1_Control_sample_15
Group_1_Control_sample_16
Group_1_Control_sample_17
Group_1_Control_sample_18
Group_1_Control_sample_19
Group_1_Control_sample_20
Group_3_Patient2_sample_25
Group_3_Patient2_sample_26
Group_3_Patient2_sample_27
Group_3_Patient2_sample_28
